# Population-based paediatric respiratory infection surveillance: a prospective inception feasibility cohort study

**DOI:** 10.1186/s40814-018-0371-8

**Published:** 2018-12-10

**Authors:** Emma C. Anderson, Suzanne Ingle, Peter Muir, Charles R. Beck, John P. Leeming, Joanna Kesten, Christie Cabral, Alastair D. Hay

**Affiliations:** 1 Centre for Academic Child Health, Population Health Sciences (PHS), Bristol Medical School (BRMS), University of Bristol, 1-5 Whiteladies Road, Bristol, BS8 1NU UK; 2 NIHR Health Protection Research Unit in Evaluation of Interventions (HPRU) and Centre for Academic Primary Care (CAPC), PHS, BRMS, University of Bristol, Canynge Hall, 39 Whatley Road, Bristol, BS8 2PS UK; 3 Public Health England, Bristol, UK; 4 HPRU, PHS, BRMS, University of Bristol, Canynge Hall, 39 Whatley Road, Bristol, BS8 2PS UK; 5 Field Service, National Infection Service, Public Health England, 2 Rivergate, Temple Quay, Bristol, BS1 6EH UK; 6 Bristol Centre for Antimicrobial Research and Evaluation (BCARE), Pathology Sciences, North Bristol NHS Trust, Bristol, BS10 5NB UK; 7 The National Institute for Health Research Collaboration for Leadership in Applied Health Research and Care West (NIHR CLAHRC West) at University Hospitals Bristol NHS Foundation Trust, Bristol, UK

## Abstract

**Background:**

There is a need to reduce unnecessary general practitioner (GP) consultations and improve antibiotic stewardship in primary care. Respiratory tract infections (RTIs) in children are the most common reason for consulting and prescribing. Most RTI research is conducted at the point of consultation, leaving a knowledge gap regarding the population burden of RTIs.

**Methods:**

Community-based, online prospective inception cohort study with nested qualitative study, to evaluate the feasibility and acceptability of collecting RTI symptom and microbiological data from children recruited prior to RTI onset.

**Results:**

Parents of 10,310 children were invited. Three hundred thirty-one parents of 485 (4.7%) children responded and completed baseline data. Respondents were less socioeconomically deprived (*p* < 0.001) with younger (median ages 4 vs. 6 years, *p* < 0.001) children than non-respondents. The same parents reported 346 RTI episodes in 259 children, and 305 RTIs (in 225 children) were retained to parent-reported symptom resolution. Restricting analyses to the first RTI episode per family (to account for clustering effects), parents fully completed symptom diaries for 180 (87%) of 192 first illness episodes. Research nurses conducted home visits for 199 RTI episodes, collecting complete (symptomatic) swab sets in 195 (98%). Parents collected 194 (98% of 199 possible) symptomatic (during the nurse visit) and 282 (92% of 305 possible) asymptomatic swab sets (on symptom resolution, no nurse present). Interviews with 30 mothers and 11 children indicated study acceptability.

**Conclusions:**

Invitation response rates were in the expected range. The high retention and qualitative evidence suggest that community-based paediatric syndromic and microbiological surveillance research is feasible.

**Electronic supplementary material:**

The online version of this article (10.1186/s40814-018-0371-8) contains supplementary material, which is available to authorized users.

## Key messages

- There is a need to reduce unnecessary GP consultations and improve antibiotic stewardship in primary care, with respiratory tract infections (RTIs) in children being an important target for intervention since they are the most common reason for consulting and prescribing.
- Most RTI research is conducted at the point of consultation, leaving a knowledge gap regarding the population burden, illness duration and microbiological cause of community RTIs, and factors affecting consulting behaviour.
- This study demonstrates the feasibility of recruiting and following up children with RTIs in the community, including collecting microbiological and symptom severity and duration data.
- Future studies should aim to improve sample representativeness.

## Introduction

Respiratory tract infections (RTIs) are the most common problem managed by primary care, with the majority occurring in children [1]. Primary care resources are overstretched [2] and antibiotics are over-prescribed [3, 4], increasing the global threat of antimicrobial resistance (AMR) [5, 6]. Diagnostic uncertainty as well as a fear of ‘missing the sick child’ contributes to antibiotic prescribing [7, 8].

Community-based RTI research [9–11] and influenza surveillance [12–16] are frequently conducted at the point of consultation with primary care. While this approach supports national and international surveillance aims, the epidemiological burden of community-based RTIs at the local population level is not captured. Therefore, the population-based incidence, microbiological cause, clinical course, and both the proportion of the population incidence and factors affecting consultation frequently remain unknown. Although a recent study shed some light on the RTI ‘clinical iceberg’ for adults [4], it was based on retrospective self-report and excluded children. A community RTI surveillance initiative is underway in Germany, which gathers routine swabs from infected pre-school children [17]. To our knowledge, no surveillance programme has collected symptomatic data prospectively and in real time nor swabs during both symptomatic and subsequent asymptomatic phases (and across a range of children’s ages) and certainly not in the UK.

Enhanced knowledge of paediatric RTIs (including the microbiological cause) in the community is hypothesised to have wide-ranging benefits. First, it would provide the ability to compare consultations and prescribe against the true denominator of symptomatic people in the community (as opposed to the registered population or prescription numbers). This would lead to an improved understanding of the consultation burden and trends in antibiotic prescribing. For example, we know that there was a recent drop (6%) in primary care prescribing of all antibiotics in England between 2014 and 2015 [18]. Since this is based on the denominator of total prescriptions, it remains unknown if the reduction is because fewer people became symptomatic, fewer presented to primary care, fewer antibiotics were prescribed to those presenting, or a combination thereof. Community surveillance could provide enhanced knowledge of the factors leading to prescribing which in turn would help in the development of interventions to support better use of the National Health Service (NHS) by the public and prescribing by clinicians.

Second, understanding the microbiological cause of the true burden of illness (not just those that present to the NHS) could be useful for the prioritisation of vaccine and antiviral drug development.

Third, if conducted in real time, community surveillance data could aid healthcare resource planning by providing early warning of the increasing incidence of an infection, such as respiratory syncytial virus, that could lead to increasing hospital admissions for infants with bronchiolitis.

Finally, real-time surveillance could reduce diagnostic uncertainty for clinicians, by indicating likely diagnoses for patients that match the symptom profiles of current locally circulating infections.

Therefore, the aim of this study was to assess the feasibility and acceptability of recruiting and retaining a community cohort of children with RTIs into an online study collecting prospective symptom and microbiological data. We will report the natural history of the illnesses, and their microbiology, in separate publications.

## Methods

### Design and recruitment

This was a prospective, community-based, feasibility inception cohort study of children and their parents/carers (hereafter ‘parents’), with a nested parent and child qualitative evaluation. As a detailed description of study methods is presented elsewhere [19], we present a brief summary below.

We purposively recruited GP surgeries that represented a range of areas of deprivation [19] in a large city in the South West of England. Children and their parents were recruited between 26 February and 1 July 2016. Invitations and recruitment were staggered over 14 and 18 weeks, respectively. Recruitment was initiated by GP letter (with consent form, information leaflet and return envelope), sent ‘to the parent/carer’ of every registered immunocompetent child aged ≥ 3 months and < 15 years (15-year-olds were excluded to avoid anyone turning 16 during the study and therefore requiring further consent), meaning some families received multiple mail packs (one per child). Supplementary efforts were made to boost responses to letters including repeat mail-outs, snowball recruitment, GP practice text messages and website promotion.

On receipt of a valid completed postal consent form, an administrator telephoned to confirm eligibility and the parent was emailed a link to complete an online baseline questionnaire for each eligible child in the household. Cohort enrolment (and study participation) was defined as receipt of written consent and completion of the online baseline questionnaire.

### Data sources/measurement

**Fig. 1:**
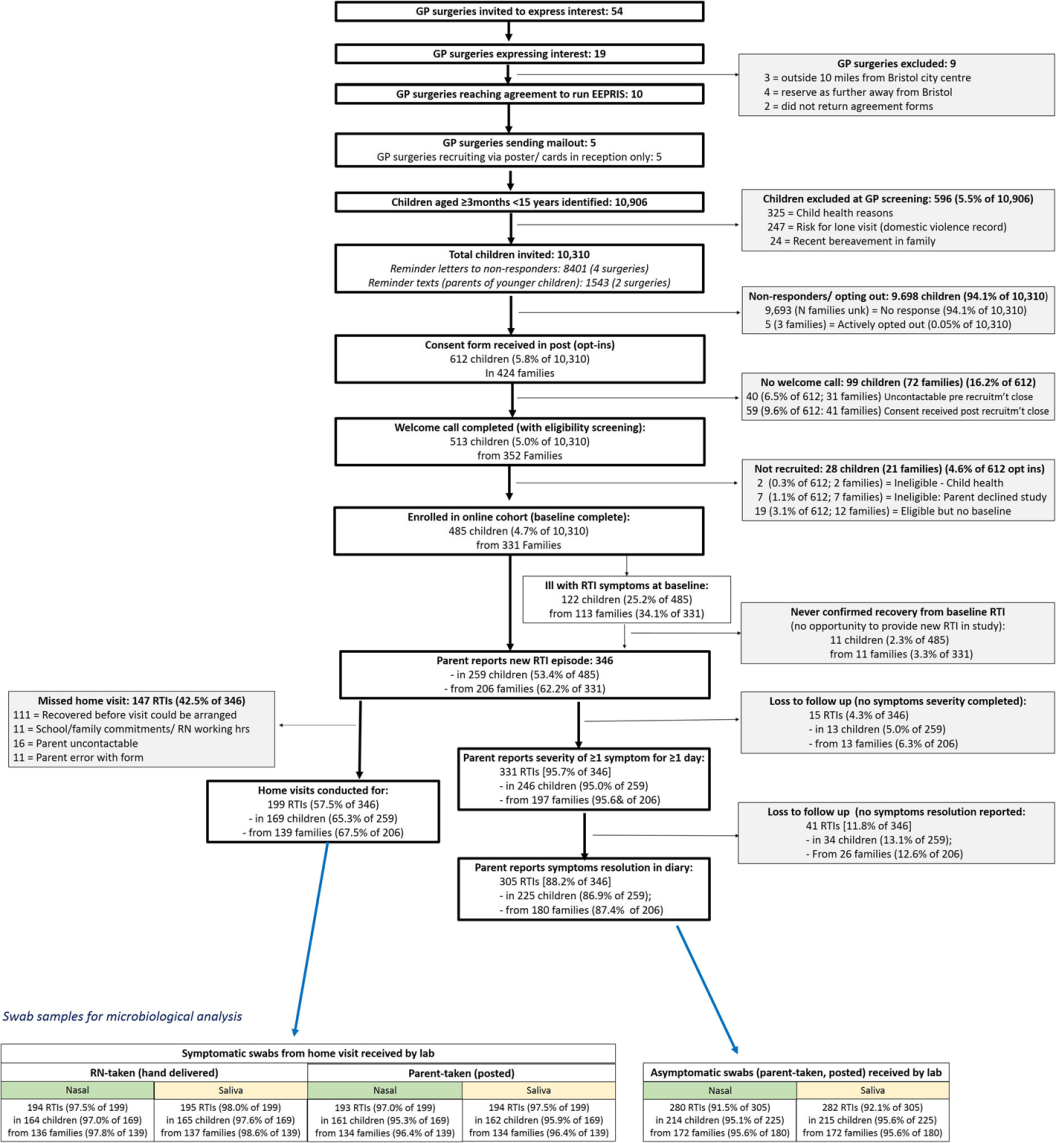
Data collection flowchart

Main data collection processes are shown in Fig. 1 and presented in detail elsewhere [19]. Baseline questionnaire completion was followed by a weekly email per eligible child to check presence/absence of new RTI symptoms (at least one of the following: runny/blocked nose, earache/ear discharge, cough, sore throat, chesty symptoms) in the previous week*.* RTI symptoms present at baseline were not included since symptom duration would have to be estimated retrospectively. On confirming the presence of a new RTI, parents completed a daily symptom diary (until symptom resolution) and an illness impact questionnaire, online. Symptomatic nasal and saliva samples were taken during the home visit: first, by the parent (using dry collection tubes) guided by an instruction leaflet provided (see Additional file 1), aiming to test parent swab-taking without nurse support. Parents were asked to post the swabs to the laboratory after the visit (postal kit provided). Second, the research nurse took similar swabs but stored them in preservative broth and took these to the laboratory in a cold storage box on the same day (as a ‘gold standard’ swab method for comparison). On subsequently confirming symptom resolution, parents were prompted to take a final set of (asymptomatic) samples (dry tubes) to post to the laboratory using equipment (and specimen postal package) left by the nurse at the home visit (or posted to the parent if the home visit was missed). While nasopharyngeal swabs are widely regarded as the optimal upper respiratory tract sampling method, we ruled them out as they are painful, uncomfortable, children will not tolerate repeat sampling and there is little chance of this being done by parents in the community.

At RTI resolution, parents were invited to (i) opt in/out of reporting further RTI episodes and (ii) respond to a questionnaire rating the user-friendliness of online data collection. Parents received a £15 gift voucher for each child that contributed a full (or nearly full) set of RTI data online.

No new RTIs were included after July 2016, and data collection ended on resolution of final RTIs in August 2016. Primary care medical notes were reviewed (medical history, relevant consultations and prescriptions) in October 2016 for all children who contributed data on at least one RTI.

A subsample of consenting parents was invited to be interviewed regarding the acceptability of the study. Parents were purposefully selected for face-to-face interview based on socio-economic status (index of multiple deprivation [IMD] decile using home postcode), age of the parent’s child (< or ≥ 7 years) and whether RTI symptoms were reported within the study, aiming for a spread of study participants across SES deciles and age of their children. If aged ≥ 7 years, the participating children of the parents who were interviewed were invited to interview about study acceptability along with their parent. Parents received a £5 gift voucher as a thank you for participating in an interview.

A Patient and Public Involvement team of eight parents advised on recruitment documents and processes, to maximise accessibility to the study.

### Outcomes

Primary outcomes were recruitment and retention rates. Recruitment was defined as the number of new RTIs reported per total recruited children/families within the cohort; retention was the number of complete data sets, defined as the number of RTIs retained to symptom resolution (parent-reported two consecutive symptom-free days), compared with the number of children/families within the cohort.

Secondary outcomes were (i) representativeness of study sample, comparing gender, age and IMD (from home postcode) with non-responders; (ii) data completion, including home visits, microbiological samples received by the laboratory and notes review data completed for those contributing RTI symptoms; and (iii) acceptability of study processes assessed via interviews supplemented by a questionnaire.

### Sample size and data analysis

Based on published research using similar methods [20], we predicted a 5% response to invitation and aimed to recruit until we achieved 300 incident RTIs [16]. We used descriptive statistics to summarise recruitment, retention, data completion and baseline characteristics, with means and standard deviations (SDs) for normally distributed continuous variables, medians and interquartile ranges (IQRs) for non-normally distributed continuous variables and percentages for categorical variables. Cohort representativeness was assessed by comparing age, gender and IMD of invited children with those enrolled using appropriate tests: *χ*^2^ tests for categorical variables and Wilcoxon rank-sum tests for non-normally distributed continuous variables. Qualitative data were analysed using the framework method [21].

## Results

### Recruitment

**Fig. 2:**
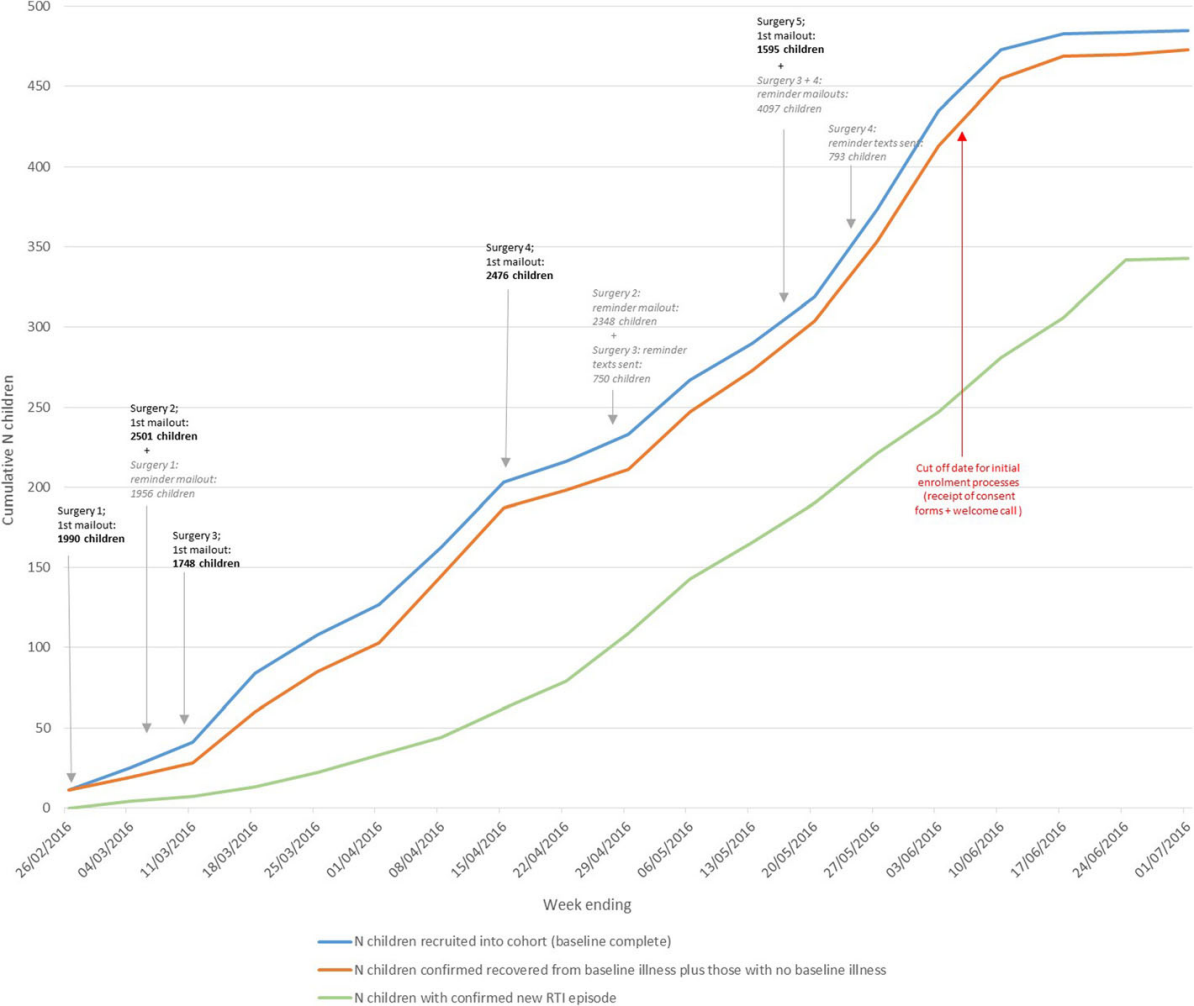
Recruitment Graph

Five surgeries agreed to participate in the study and sent letters inviting the parents of 10,310 children to participate (Fig. 1). Reminder letters were sent to non-responders from four of the five practices (Fig. 2, none to those from the fifth due to limited time) and texts to non-responders from two (not all practices had this facility). Due to meeting study targets (300 RTIs) via higher than predicted RTI reporting per family as recruitment progressed, an additional five GP practices displayed posters only (did not send invitation letters). Of the five surgeries sending invitation letters, surgery 1 (IMD 3) and surgery 2 (IMD 1) had lower consent form return rates (3.1% and 3.5%, respectively) compared to surgeries 3 (IMD 8) and 5 (IMD 9) at 7.4% and 8.3%, respectively. Surgery 4 (IMD 2) had a 6.9% recruitment rate.

Of the 612 consent forms received, 59 (9.6%) were not enrolled due to receiving these after the recruitment cutoff date (Fig. 1). The welcome call identified two ineligible children who were not recruited into the cohort. Four hundred eighty-five children (4.7% from 10,310 invitations) were recruited (baseline completed) from 331 families. One hundred twenty-two (25.2%) reported RTI symptoms at baseline.

### Participants

When asked how they first heard about the study, 318 (96.0% of 331) parents reported ‘GP letter’, three ‘GP text’, five ‘poster/card in surgery’, four ‘word of mouth’ and one reported ‘university website’. Seven participants were recruited without having been sent an invitation letter: one from a surgery that sent no mail-out (poster advertisement only) and six from surgeries that did. All other respondents received a letter invitation from their surgery (even if they first heard about it elsewhere).

Participating parents were mainly mothers (91%), with an average age of 39 (IQR 35–45) years, with most (88%) self-identifying as ethnically white (Table 1). Fifty-four percent reported working part-time and 23% full-time. Parents were highly educated, with 85% reporting having a first or higher degree and 20% reporting having some medical or nursing training. Most (77%) households had two resident adults with 42% reporting one and 45% two resident children (comparable to national averages) [16]. Half the participating children were female, and the cohort had a median age of 4 (IQR 2–8) years.

**Table 1:** Baseline characteristics of the cohort

		Number	(Percentage)
Parent characteristics (*N* = 331)			
Gender	Missing	3	(0.91)
	Female	301	(90.94)
	Male	27	(8.16)
Age	Missing	13	(3.93)
	Median years (IQR)	39 (35–45)	
Ethnicity	Missing	14	(4.23)
	Asian	7	(2.11)
	Black	10	(3.02)
	Mixed	8	(2.42)
	White	290	(87.61)
	Other	2	(0.60)
Employment	Missing	14	(4.23)
	Full-time parent/care-giver	45	(13.60)
	In full-time education	3	(0.91)
	Not currently employed	13	(3.93)
	Working full-time	77	(23.26)
	Working part-time	179	(54.08)
Education	Missing	14	(4.23)
	Up to GCSEs/GCEs/‘O’ levels or equivalent	14	(4.23)
	‘A’ levels/NVQs/GNVQs or equivalent	19	(5.74)
	First degree/diploma/HNC/HND or equivalent	165	(49.85)
	Higher degree (e.g. MSc, PhD) or equivalent	116	(35.05)
	No official qualification	3	(0.91)
Do you have any medical training?	Missing	14	(4.23)
	No	251	(75.83)
	Yes	66	(19.94)
Household characteristics (*N* = 331)			
Bedrooms per person in household	Median (IQR)	0.88 (0.67–1)	
Smoker in house	No	292	(88.22)
	Yes	25	(7.55)
No. of adults (aged 16+) living in child’s main home	Missing	14	(4.23)
	0	1	(0.30)
	1	29	(8.76)
	2	253	(76.44)
	3	23	(6.95)
	4+	11	(3.32)
Age of adults in home	Median years (IQR)	38.5 (35–44)	
Total no. of children in home	Missing	14	(4.23)
	1	140	(42.30)
	2	149	(45.02)
	3	24	(7.25)
	4	3	(0.91)
	5	1	(0.30)
Child-level characteristic (*N* = 485)			
Age	Median years (IQR)	4 (2–8)	
Gender	Female	245	(50.52)
	Male	240	(49.48)
Ethnicity	Asian	13	(2.68)
	Black	16	(3.30)
	Mixed	32	(6.60)
	White	419	(86.39)
	Other	5	(1.03)
Does child attend school or day care?	School	261	(53.81)
	Day care (3–5 days per week)	78	(16.08)
	Day care (1–2 days per week)	81	(16.70)
	No (or not that I am aware of)	65	(13.40)

Participating children were comparable to non-respondents with respect to gender but were younger (*p* < 0.001) and residing in lower deprivation index areas (*p* < 0.001, Table 2).

**Table 2:** Representativeness of children in cohort

	Invited, not enrolled (*N* = 9832)	(Percentage)	Enrolled (*N* = 485)	(Percentage)	Chi-squared *p* value
Age, years median (IQR)	6 (3–10)		4 (3–7)		< 0.001*
Female, *N* (%)	4824	(49.1)	245	(50.5)	0.53
IMD decile, *N* (%):					< 0.001
1 (Most deprived)	2301	(23.4)	42	(8.7)	
2	889	(9.1)	44	(9.1)	
3	1316	(13.4)	43	(8.9)	
4	1151	(11.7)	59	(12.2)	
5	339	(3.5)	16	(3.3)	
6	352	(3.6)	46	(9.5)	
7	960	(9.8)	65	(13.4)	
8	1640	(16.7)	109	(22.5)	
9	395	(4)	25	(5.2)	
10 (Least deprived)	485	(4.9)	36	(7.4)	

Four children in not-enrolled group had missing data for age and IMD decile. Seven children in enrolled group were not in original mail-out invitation list

*Wilcoxon rank-sum *p* value

### New RTIs and study retention (primary outcomes)

Parents reported 346 RTIs in 259 (53%) of children from 206 families. Of these, symptom duration data were complete in 305 (88%, Fig. 1). This completion rate was similar when considering first RTI per child (87%) and first RTI per family (87%). More educated parents were more likely to complete symptom duration data compared to less educated parents, but no other child or parent factors were associated with symptom data completion (Table 3). Seventy (27% of 259) children from 62 families contributed data on more than one RTI. Thirty-four (7%) children withdrew, of whom 28 withdrew after confirming symptom resolution for their first RTI.

**Table 3:** Characteristics at baseline restricting to first RTI episode within a family, according to whether or not they started and completed symptom diaries, *N* parents = 206, *N* children = 259

			Completed symptoms data?				
	*N*	(%)	*N*	(%)	*N*	(%)	*p* value
Parent characteristics (*N* = 206)			No (*n* = 26)		Yes (*n* = 180)		
Gender							0.19
Missing	2	(0.97)					
Female	193	(93.69)	26	(100)	167	(93.82)	
Male	11	(5.34)	0	(0)	11	(6.18)	
Age, years* (median, IQR)	38 (34–43)		36 (34–41)		38 (34–43)		0.33
Missing	9	(4.37)					
Ethnicity							0.22
Missing	9	(4.37)					
Asian	5	(2.43)	0	(0)	5	(2.91)	
Black	5	(2.43)	2	(8.00)	3	(1.74)	
Mixed	5	(2.43)	1	(4.00)	4	(2.33)	
White	182	(88.35)	22	(88.00)	160	(93.02)	
Employment							0.64
Missing	9	(4.37)					
Full-time parent/care-giver	34	(16.50)	4	(16.00)	30	(17.44)	
In full-time education	2	(0.97)	0	(0)	2	(1.16)	
Not currently employed	8	(3.88)	2	(8.00)	6	(3.49)	
Working full-time	41	(19.90)	7	(28.00)	34	(19.77)	
Working part-time	112	(54.37)	12	(48.00)	100	(58.14)	
Education							0.02
Missing	9	(4.37)					
Up to GCSEs/GCEs/‘O’ levels or equivalent	8	(3.88)	4	(16.00)	4	(2.33)	
‘A’ levels/NVQs/GNVQs or equivalent	9	(4.37)	2	(8.00)	7	(4.07)	
First degree/diploma/HNC/HND or equivalent	107	(51.94)	11	(44.00)	96	(55.81)	
Higher degree (e.g. MSc, PhD/equivalent)	71	(34.47)	8	(32.00)	63	(36.63)	
No official qualification	2	(0.97)	0	(0)	2	(1.16)	
Do you have any medical training?							0.22
Missing	9	(4.37)					
No	155	(75.24)	22	(88.00)	133	(77.33)	
Yes	42	(20.39)	3	(12.00)	39	(22.67)	
Child characteristics (*N* = 259)			No (*n* = 34)		Yes (*n* = 225)		
Age, years (median, IQR)	3 (1–6)		3 (1–6)		3 (1–6)		0.78
Gender							0.93
Female	143	(55.21)	19	(55.88)	124	(55.11)	
Male	116	(44.79)	15	(44.12)	101	(44.89)	
Ethnicity							0.54
Asian	10	(3.86)	2	(5.88)	8	(3.56)	
Black	6	(2.32)	1	(2.94)	5	(2.22)	
Mixed	12	(4.63)	3	(8.82)	9	(4.00)	
White	231	(89.19)	28	(82.35)	203	(90.22)	
Other							
Does child attend school?							0.56
No (or not that I am aware of)	149	(57.53)	18	(52.94)	131	(58.22)	
Yes	110	(42.47)	16	(47.06)	94	(41.78)	
Does child attend day care regularly?							0.29
No (or not that I am aware of)	42	(28.19)	7	(38.89)	35	(26.72)	
Yes (1–2 days per week)	59	(39.60)	8	(44.44)	51	(38.93)	
Yes (3–5 days per week)	48	(32.21)	3	(16.67)	45	(34.35)	

Research nurses conducted home visits in 199 (58%) of the 346 RTI episodes, in 169 (65% of the 259) children from 139 (67% of the 206) families who reported RTIs in the study (Fig. 1). The majority (111) of the 147 (42%) RTIs missing home visits were due to the child recovering before the visit could be arranged. Over 97% of all the symptomatic swabs due were received by the laboratory, whether taken to the laboratory by the nurse or posted by parents, and over 90% of asymptomatic swabs due reached the laboratory by post (Fig. 1). Primary care notes were reviewed by the study research nurses for all 346 (100%) children contributing one or more RTIs to the study.

### Qualitative and quantitative acceptability evaluation

Parent interviews suggested that the welcome phone call provided a useful opportunity to ask questions and for parents to check their understanding of what the research involved. Parents approved of the personal aspect of speaking to a friendly member of the study team. Most parents would have been happy not to receive a welcome phone call as the information sheet was generally viewed as sufficient. Parents approved of weekly emails arriving in the evening when they had time to respond but noted that they did not allow them to record symptoms which appeared and resolved between emails. Parents were happy with the time commitment required and with providing information for more than one RTI. While many parents found identifying symptoms straightforward, several described confusion around whether to report very mild symptoms to the study. Parents found the daily symptom record clear and easy to complete. Parents could choose the day and time for research nurse visits, which they found convenient. There were mixed views on the clarity of swab instructions, and several parents described the mouth swabs as more difficult to use than the nasal. Parents described some anxiety about taking swabs. Although parents felt they would have been able to take samples without the nurse, they felt more confident with the nurse present. Though the design was aimed to minimise this (to assess feasibility of parent swab-taking), nurses provided advice to parents on taking the swabs. Observing the nurse take swabs helped parents learn how to take subsequent samples. The majority of children found the swabs acceptable and enjoyed the nurse visit, though some younger children (infants and toddlers) disliked the swabs and some older children described the nasal swab as uncomfortable. See Table 4 for a summary of themes and illustrative participant quotations.

**Table 4:** Acceptability themes and quotes from parent interviews

Theme	Quote
Welcome telephone call	*They seemed to be able to answer any questions about, I was concerned about the frequency of what I would have to do as a parent, so how much involvement there was initially and she was quite good at answering all my questions (Interview 18, IMD decile 8, child age 3 years 6 months)*
	*For me personally, the fact that I’d read everything and understood it, and not needed to contact… I felt that she could have done without it.*
	Interview 8, IMD decile 8, child age 3 years 4 months
Daily symptom record	*Having been through a cycle [of data collection for one illness episode] it was clear that it did not really impact in our lives in any kind of negative way so there was no reason to pull out.*
	Interview 25, IMD decile 8, child age 1 year 7 months
	*It was fairly straightforward and I think because I knew what I was doing, especially after the first day of filling in the questionnaire you kind of remember maybe a bit more clearly in relation to what the list of symptoms were kind of ‘yes, he’s got that today, he did not have that yesterday, I must remember to put that down’…*
	Interview 25, IMD decile 8, child age 1 year 7 months
Study communication	*I have initiated contact to say they are symptomatic because, to my mind, my slight issue about the study is this whole issue about the weekly contact, and you can literally have missed an entire illness episode.*
	Interview 22, IMD decile 8, child age 4 years 2 months
	*The emails tend to come through on an evening, as well, which is brilliant, because in the evening is generally when I have got the time to be dealing with it.*
	Interview 3, IMD decile 1, Child age 8 years 6 months
Research nurse visit and swab taking	*It [swab instructions] was just a lot of stages to it. Especially while you have got a toddler there waiting to have something put up their nose or something and you are like, ‘Hang on a minute. I have just got to read the instructions.’*
	Interview 15, IMD decile 4, child age 3 years 0 months
	*There was that level of slight anxiety of what do I have to do and am I getting this right and going back to the instructions.*
	Interview 7, IMD decile 10, child age 9 years 10 months
Child response	*I mean from what I was seeing she was feeling a little bit important, you know it’s something special a bit, she does not normally do it so it’s actually very nice and we are saying its helping research and she feels special about you know.*
	Interview 13, IMD decile 6, child age 10 years 9 months
	*When we took her for the sample from the nose or the saliva, yeah she was just crying.*
	Interview 21, IMD decile 8, child age 1 year 3 months

In rating how user-friendly they found the study surveys, 154 (86%) of 180 parents responded ‘very’ or ‘quite’, 19 (11%) responded ‘fairly’ and 7 (4%) responded ‘not really’ or ‘not at all’. Free text responses fell into three categories: (i) complimentary, (ii) indicated differing preferences for (more/fewer) prompts or (iii) referred to technical issues (resolved during the study or identified to be resolved in a future study). When asked if they would have preferred paper rather than online questionnaires, almost all (98%) of parents’ responses were ‘no’.

## Discussion

### Summary of main results

GP surgery and participant recruitment, along with high retention of children and parents, suggests that population-based paediatric RTI surveillance, with detailed symptom data collection and microbiological sampling, is feasible and acceptable. Most participants were recruited via GP invitation letter, with supplementary efforts (posters, contact cards, GP texts and UoB website) making little difference to recruitment. A higher than anticipated proportion of the cohort (25%) reported RTI symptoms at baseline, and a higher than anticipated proportion (28%) reported more than one RTI per child, even without incentives to report more than one RTI per child. This latter finding gives some suggestion that, while study participants had limited time in which to contribute data, longer-term or ongoing surveillance may also be feasible and acceptable. Had we asked parents to follow illnesses in their children for a lot longer, however, we may have seen more attrition.

Successful microbiology data collection indicates that (i) both nasal and saliva swab collection are feasible and (ii) parents reliably returned samples in the post when requested, even for samples (asymptomatic) which were prompted to be taken on questionnaire completion only (without a nurse present). Even though nurses were advised to take swabs after parents (to assess feasibility of independent swab taking), some parents reported receiving nurse help. Results of swab sample analysis (including quality comparisons) will be presented in a separate paper.

### Strengths and limitations

There was greater than expected retention in ongoing surveillance once participants were enrolled in the study, shown by low withdrawal rates and high agreement to continue in the study after contributing a first set of RTI data. Many parents contributed RTI data for more than one child and more than one RTI in each child.

Our cohort was not socio-demographically representative of those contacted, with over-representation of highly educated parents, residence in less deprived neighbourhoods and younger children than the source population. Recent findings show that access to the internet (within the last 3 months) in UK households with children increased from 95 to 100% between 2012 and 2018 [22], which makes the online methodology unlikely to be a cause of the lack of uptake from areas with lower deprivation decile. While the issue of recruiting hard-to-reach populations, as found in many studies, is likely due to multiple factors, one problem with respondent recruitment in more deprived areas has been shown to be high levels of ineligible addresses and non-contacts (rather than refusal to participate) [23]. It is possible that our main recruitment method (invitation via mail-out) met with this problem. It is not clear whether retention would be as high in a more representative sample. Invitation and enrolment were staggered, with children from the final invitation batch having a very limited time to contribute RTI data before study closure. There is no adjustment for time at risk of developing illness for any individual in this cohort, as this is a feasibility study with simple descriptive results, though future research could build this into analysis.

The aim was to conduct the study over winter months, rather than the spring/summer data collection achieved, which may have reduced the number of RTIs and led to confusion between RTI and allergic symptoms. Conducting home visits before a child recovered was also a challenge because of the design of the online data collection forms. The email checking for new RTI symptoms was sent each Sunday only, and the database did not allow parents to initiate a *new* RTI symptom questionnaire between weekly emails. This meant that children who developed new RTI symptoms at the beginning of the week could recover before the study team were alerted to the new RTI to arrange a home visit.

Our qualitative finding that some parents were unsure about reporting very minor symptoms could be interpreted as suggesting a slight bias towards reporting more severe illnesses. However, in its low threshold for reporting RTI, our study design is likely to have captured a greater number of milder illnesses than other research to date (i.e. asking parents to report mostly benign and very common symptoms, to which parents may not otherwise have paid any attention).

### Comparison with existing literature

To our knowledge, this is the first study to use online methods to collect detailed symptom and microbiological data from children with RTIs in the community (i.e. population-level surveillance data as opposed to a consulting population) and (in contrast to McNulty et al. [4]) to gather RTI data at the time of the infection rather than retrospectively. Response rates to the study invitation was low but in line with that observed in a study using similar recruitment methodology, albeit investigating the effects of a hand-washing intervention in households, not exclusively children [24]. Data collection was close to real time, rather than retrospective recall, which is a strength of the current study compared to previous research into the RTI ‘clinical iceberg’ (conducted on adults) [25].

### Implications for research and/or practice

Future research should be longer-term to enable exploration of year-on-year changes in the seasonality of community RTI rates, especially investigating for changes in microbiology and symptoms. Future randomised studies could test the real-time presentation of locally relevant symptom/microbiological data as an intervention to support primary care antibiotic prescribing. Sample representativeness could be improved by increasing recruitment efforts in lower deprivation index areas, one example being to use stratified sampling methods such as weighting more invitations from practices in areas of lower deprivation index (according to our findings, three or four times as many letters from a practice in deprivation decile 1 compared to those in decile 10 may be warranted) and targeting older children.

Methodological recommendations for a future definitive study include the following: (i) planning to account for RTI symptoms at baseline in a sizable proportion of community-recruited participants (by collecting data on these illnesses or accounting for resolution of baseline illnesses in data collection planning), (ii) allowing questionnaire access for new RTIs in between weekly prompts, (iii) removing the mandatory welcome call (while offering optional telephone contact for participant queries) and using purely online consent followed immediately by baseline questionnaire (making the recruitment pathway more efficient for more timely commencement of data collection and potentially avoiding the problem of respondents not being recruited if they were unavailable via telephone) and (iv) enrolling all children to start simultaneously early in the winter season (before typical seasonal epidemics begin) and for longer durations to enhance the usefulness of data by enabling robust analysis of RTIs per child-year, a comparison of characteristics of children that developed RTI with those who did not, and to explore the seasonality of infection rates.

## Conclusions

Response rates were low, but in the expected range. With improved generalisability, the high retention and qualitative evidence suggest that community-based paediatric syndromic and microbiological surveillance research is feasible.

## Additional file

Additional file 1: Collecting samples from your child: instructions for parents. (PDF 2249 kb)

## References

[1] Hay AD, Heron J, Ness A (2005). The prevalence of symptoms and consultations in pre-school children in the Avon Longitudinal Study of Parents and Children (ALSPAC): a prospective cohort study. Fam Pract.

[2] The Information Centre - Primary Care Statistics. 2006/07 UK General Practice Workload Survey. Government Statistical Service, ed.; 2007.

[3] Ashworth M, Charlton J, Ballard K (2005). Variations in antibiotic prescribing and consultation rates for acute respiratory infection in UK general practices 1995–2000. Br J Gen Pract.

[4] McNulty CA, Nichols T, French DP (2013). Expectations for consultations and antibiotics for respiratory tract infection in primary care: the RTI clinical iceberg. Br J Gen Pract.

[5] Domin MA (1998). Highly virulent pathogens--a post antibiotic era?. Br J Theatre Nurs.

[6] O'Neill J. Antimicrobial resistance: tackling a crisis for the health and wealth of nations. The Review on Antimicrobial Resistance; 2014.

[7] Teixeira Rodrigues A, Roque F, Falcao A (2013). Understanding physician antibiotic prescribing behaviour: a systematic review of qualitative studies. Int J Antimicrob Agents.

[8] Cabral C, Lucas PJ, Ingram J, et al. “It’s safer to ...” parent consulting and clinician antibiotic prescribing decisions for children with respiratory tract infections: an analysis across four qualitative studies. Soc Sci Med 2015;136–137:156–64. 10.1016/j.socscimed.2015.05.027. 10.1016/j.socscimed.2015.05.027 26004209

[9] Hay AD, Wilson A, Fahey T (2003). The duration of acute cough in pre-school children presenting to primary care: a prospective cohort study. Fam Pract.

[10] Harnden A, Perera R, Brueggemann AB (2007). Respiratory infections for which general practitioners consider prescribing an antibiotic: a prospective study. Arch Dis Child.

[11] Turnbull SL, Redmond NM, Lucas P (2015). The CHICO (Children’s cough) trial protocol: a feasibility randomised controlled trial investigating the clinical and cost-effectiveness of a complex intervention to improve the management of children presenting to primary care with acute respiratory tract infection. BMJ Open.

[12] World Health Organization. Global epidemiological surveillance standards for influenza. Edited by Influenza WHOWGESSf. Geneva: World Health Organization; 2013.

[13] Public health England (2014). Official Statistics: Weekly National Flu Reports.

[14] Public Health England (2014). Collection: syndromic surveillance: systems and analyses.

[15] Organization WH (2011). WHO regional Office for Europe guidance for sentinel influenza surveillance in humans.

[16] Blum CA, Nigro N, Briel M (2015). Adjunct prednisone therapy for patients with community-acquired pneumonia: a multicentre, double-blind, randomised, placebo-controlled trial. Lancet.

[17] Niedersächsisches Landesgesundheitsamt (Lower Saxony State Health Office) (2018). Acute Respiratory Diseases (ARE).

[18] Public Health England. English surveillance programme for antimicrobial utilisation and resistance (ESPAUR) Report 2016. London; 2016. Accessed 19 Sept 2017.

[19] Anderson EC, Ingle SM, Muir P (2016). Community paediatric respiratory infection surveillance study protocol: a feasibility, prospective inception cohort study. BMJ Open.

[20] Little P, Stuart B, Hobbs FD, et al. An internet-delivered handwashing intervention to modify influenza-like illness and respiratory infection transmission (PRIMIT): a primary care randomised trial. Lancet. 2015. 10.1016/S0140-6736(15)60127-1. 10.1016/S0140-6736(15)60127-1 26256072

[21] Gale NK, Heath G, Cameron E, et al. Using the framework method for the analysis of qualitative data in multi-disciplinary health research. BMC Medical Res Method. 2013;13:117. 10.1186/1471-2288-13-117. [published Online First: 2013/09/21]. 10.1186/1471-2288-13-117 PMC3848812 24047204

[22] Office for National Statistics (2018). Statistical Bulletin: Internet Access – households and individuals.

[23] Parry O, Bancroft A, Gnich W (2001). Nobody home? Issues of respondent recruitment in areas of deprivation. Crit Public Health.

[24] Little P (2011). A PRimary care trial of a website based infection control intervention to modify influenza-like illness and respiratory infection transmission: International Standard Randomised Controlled Trial Number Register.

[25] McNulty C, Nichols T, French DP, et al. Expectations for consultations and antibiotics for respiratory tract infection in primary care. Br J Gen Pract. 2013. 10.3399/bjgp13X669149. 10.3399/bjgp13X669149 PMC3693799 23834879

