# Supplementary material for: Population-based paediatric respiratory infection surveillance: a prospective inception feasibility cohort study
Source: Pilot Feasibility Stud. 2018 Dec 10;4:182. doi: 10.1186/s40814-018-0371-8 (PMC6287344; doi:10.1186/s40814-018-0371-8)
Supplement: Supplementary file 1 — Collecting samples from your child: instructions for parents. (PDF 2249 kb) [file 40814_2018_371_MOESM1_ESM.pdf]

Evaluation of Enhanced Paediatric Respiratory Infection Surveillance

Collecting EEPRIS samples from your child

The bugs (bacteria and viruses) making your child feel unwell are likely to be found in your child's nose and mouth. We can get an idea of what is in their nose by wiping the inside of their nostrils with a spongy bud (nasal swab) and in their mouth from the saliva that bathes the inside of the mouth (saliva sample).

The laboratory that you will send these samples to (using the posting kit) will be looking to see which bugs they can find. Collecting the samples correctly and getting them to the laboratory without delay is an essential part of the EEPRIS Study.

This is how you can make sure this part of the study goes well:

Preparing to collect the samples

- You might like to read these instructions all the way through first so you know what's coming! Give us a ring if you are unsure about what to do (phone number on back page)
- Ideally your child should not have had food, drink (other than water), or chewing gum for 30 minutes before taking the sample
- Try to make sure that your child does not have anything in their mouth
- Wash your hands
- Have the nasal and saliva kits ready with you together with the postal kit (blue plastic box and packaging materials)
- You will need a pen (to write the date you took the samples)

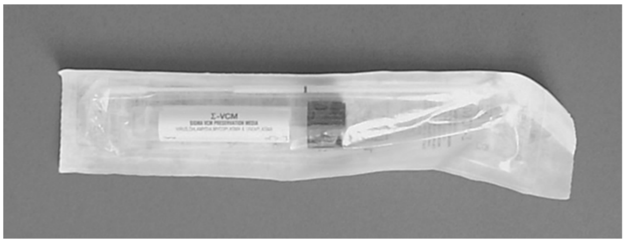

Nasal swab kit
(containing tube with red top and white straw)

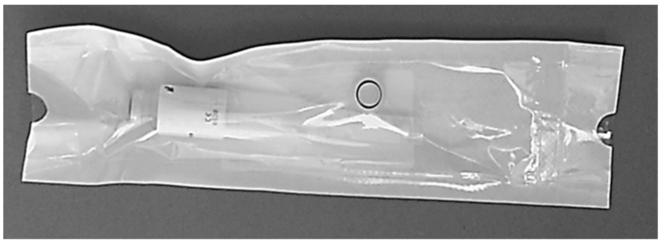

Saliva sample kit
(containing a single clear plastic tube)

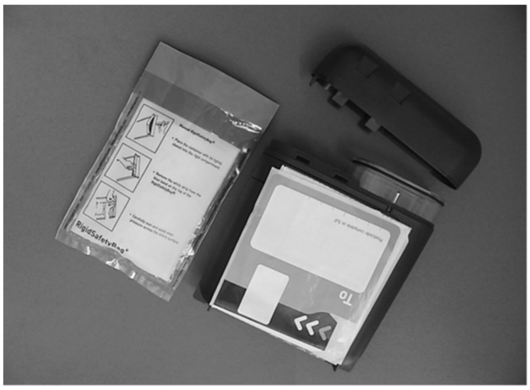

Posting kit
(containing plastic envelope and blue plastic Royal Mail box)

Sample collection instructions for parents EEPRIS Study version 5 2016-03-14

University of Bristol
NHS
Funded by NIHR
Public Health England

<u>Collecting the nasal sample</u>

1. Peel open the packet and remove the tube with the red lid

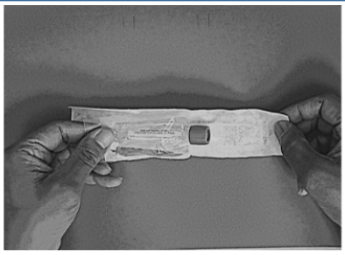

2. Unscrew the cap from the tube and keep both close by as you will need them

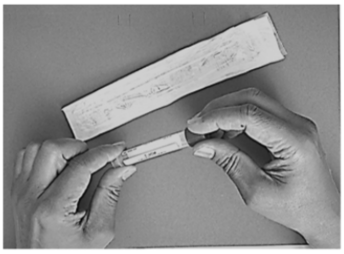

3. Remove the long plastic straw from the packet - this is the swab. Please don't touch the spongy bud on the end

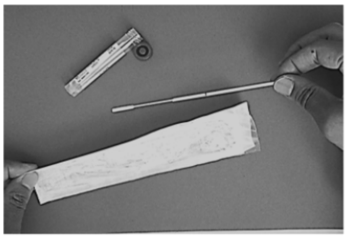

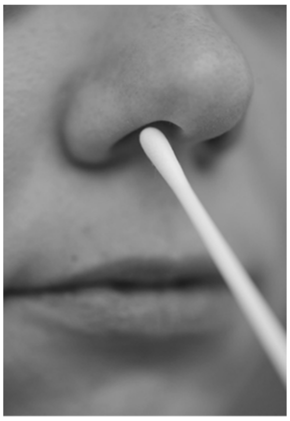

4. Gently insert the spongy end of the swab into one nostril – just inside the opening. No need to push it far up, but aim for the entire spongy end being inside the nostril (though this may not be possible for small babies)

5. Rotate the swab two or three times and then hold the swab in place for a second to absorb any liquid (snot) that may be there

6. Repeat this process in the other nostril using the same swab

7. Take the swab stick out of the nose and without letting it touch anything place it immediately into the tube from the packet

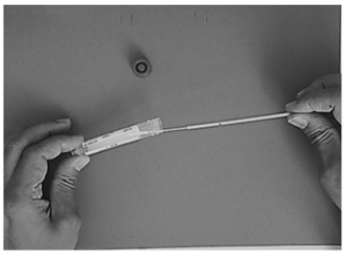

8. The straw is longer than the tube, and there is a small black mark where it will snap. With the tip still in the tube, hold the straw either side of the black mark and bend it until it snaps

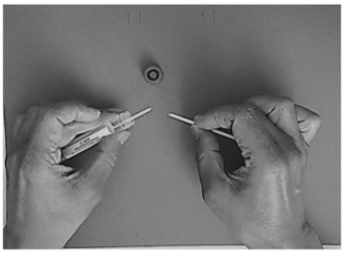

9. Screw the cap back on the tube and make sure it is tight - this will make sure the swab doesn't dry out too much. Discard the end of the straw you snapped off

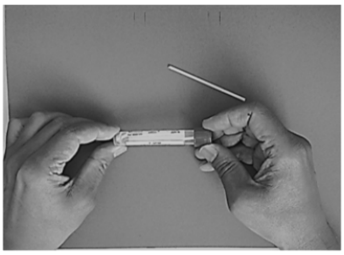

<u>Collecting the saliva sample</u>

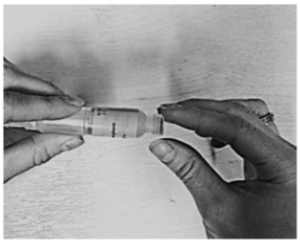

1. Peel open the packet and remove the tube. Unscrew the small cap from the end with the white label on it. Keep the cap in a safe place, you will need this again.

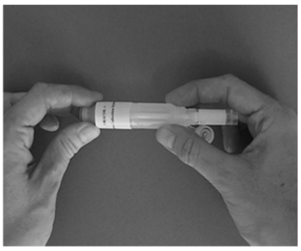

2. Hold the <u>thicker</u> (middle) part of the tube with both hands and unscrew it at this part, as shown.

   This will reveal the white plastic stick with the sponge on the end (you may need to wiggle it to pull the knobbly end out of its holder).

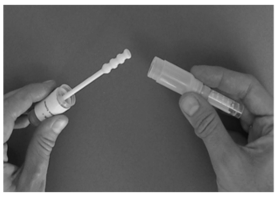

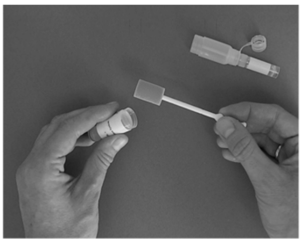

Try not to touch the sponge or let it touch anything outside of your child's mouth (we don't want any other bugs on it!)

3. Holding the white stick, pull out the sponge. Keep both parts of the tube close by as you will need them again.

4. Ask your child to open his/her mouth and then, holding the knobbly end of the stick, use the sponge to "brush" the fleshy gums at the base of their teeth. If your child wants to suck on the sponge that's fine!

5. Do this for <u>at least a minute</u>. This will help the saliva soak into the sponge. Keep the sponge there longer if you feel it could soak up more; the more saliva there is the easier it will be to find the bugs.

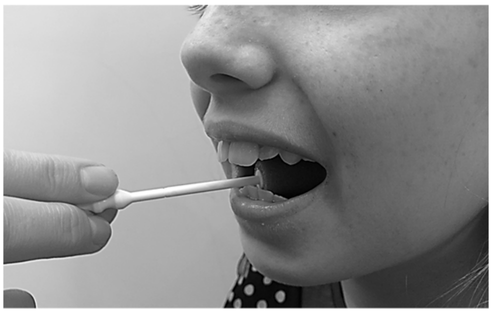

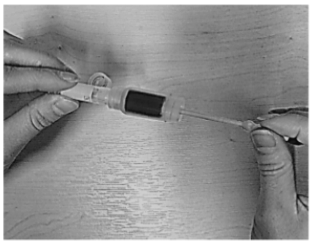

6. Once you feel the sponge isn't going to soak up any more, take it out of the mouth and without letting it touch anything place it immediately into the part of the tube <u>**without**</u> the label attached (towards the attached smaller sample pot)

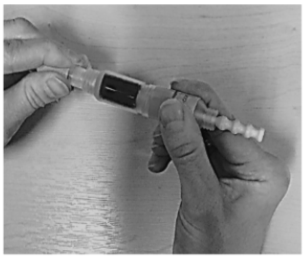

7. Replace the thicker end of the tube and screw both ends together tightly (the white stick will poke through at the thicker end).

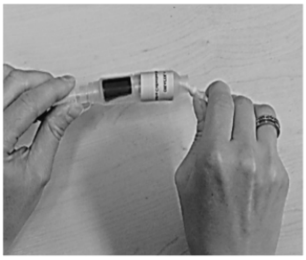

8. Snap off the white stick at the break point. (You can discard the knobbly end of the stick you have snapped off).

9. Screw the small cap back on to the end with the label on it.

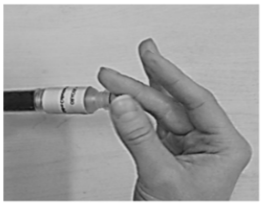

Posting the samples to the laboratory

1. Place both the saliva sample tube and the nasal swab tube inside the plastic bag provided (this will protect the tubes)

2. Seal the bag

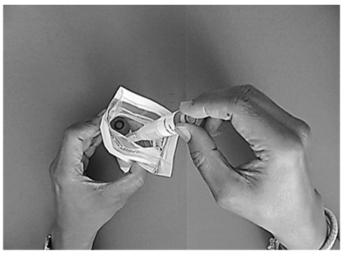

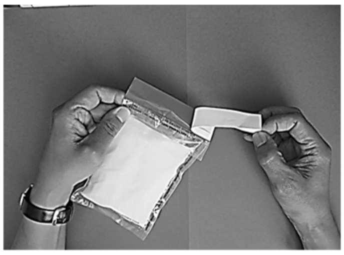

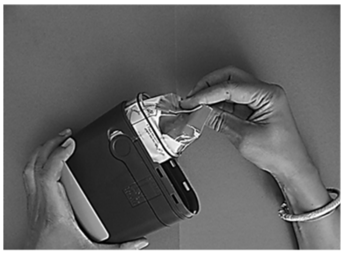

3. Put this bag in the clear plastic section of the blue hard plastic box that we have provided (we need to use all these layers of wrapping for health and safety reasons)

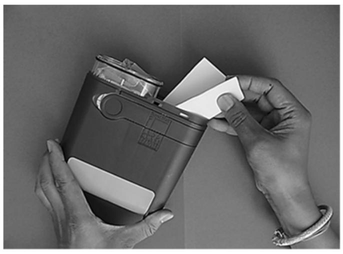

4. Write the date you took the samples on the paper form that goes with the samples and place it the other section of the blue box. (This form has your details in code so that we can make sure we know later on that the samples came from you. Each tube also has a bar code that identifies it as coming from you)

Make sure everything is inside the box - you won't be able to open it once it's closed

5. Place the lid on the blue plastic box and press it down hard until it snaps shut

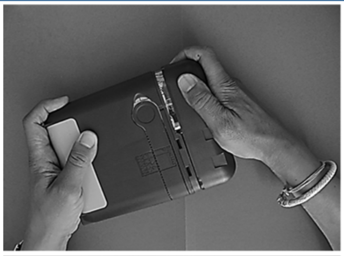

6. Peel off the back of the label and wrap it over the top of the lid and stick it down the other side (The postage has already been paid so no need to stick on stamps)

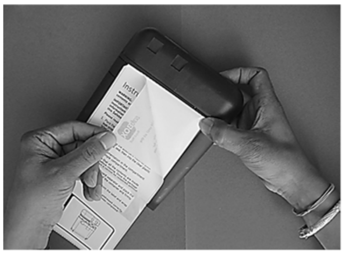

7. Please post the box in any post box as soon as you can so that it is picked up at the next collection (same day if possible)

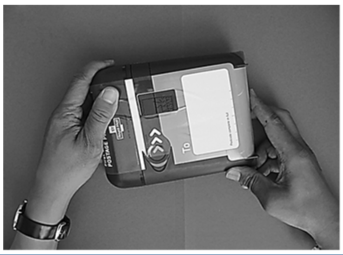

The sooner the tubes get to the laboratory, the sooner the staff can start working on them before the samples dry out

You can contact the EEPRIS Study Team:
Tel: **(0117) 33 14598**   email: **eepris-kids@bristol.ac.uk**

Let us know if you have any unused equipment (tubes, bags etc.) left after you have finished in the study, so that we can let you know what to do with them.
